# Research progress in physiological effects of resistant substances of *Urtica dioica* L. on animal performance and feed conversion

**DOI:** 10.3389/fpls.2023.1164363

**Published:** 2023-06-28

**Authors:** Yifan Zhang, Xin Zhang, Muhammad Hammad Zafar, Jinying Zhang, Jiasheng Wang, Xiang Yu, Wujun Liu, Mengzhi Wang

**Affiliations:** ^1^ Laboratory of Metabolic Manipulation of Herbivorous Animal Nutrition, College of Animal Science and Technology, Yangzhou University, Yangzhou, China; ^2^ State Key Laboratory of Sheep Genetic Improvement and Healthy Production, Xinjiang Academy of Agricultural Reclamation, Shihezi, China; ^3^ College of Animal Science, Xinjiang Agricultural University, Urumqi, China

**Keywords:** *Urtica dioica*, nettle, genetic distance, germplasm specificity, resistant material, fodderization

## Abstract

Several members of family Urticaceae are mainly found in the temperate and subtropical zones of the Northern Hemisphere and are important medicinal plants. Among them, *Urtica dioica* L. (Urticaceae) is an annual or perennial herb that has been used for feeding and medicinal purposes since long time and is the most exploited species of Urticaceae. Recently, it has received attention to be used as animal feed, as its fresh leaves fed to animals in moderate, dried, and other forms. This review details the advantages of *U. dioica* as an alternative feed in terms of germplasm specificity, nutritional composition, and feed application status. Its roots, stems, leaves, and seeds are rich in active ingredients. It has also been found to have anticancer effects through antioxidant action and promotion of apoptosis of cancer cells. In shady conditions, *U. dioica* is highly adaptable while under stressful conditions of drought; it also reduces light absorption and ensures carbon assimilation through light energy conversion efficiency. Therefore, it can be added to animal diets as a suitable feed to reduce costs and improve economic efficiency. This paper investigates the feasibility of using *U. dioica* as a feed and systematically presents the progress of research and exploitation of *U. dioica*.

## 1 Introduction

*Urtica* belongs to dicotyledonous plants, which is an important plant in terms of its fiber content (Upton, 2013). It contains irritating toxic fluids, i.e., anthranilic acid, pentazocine, histamine etc. (Wagner et al., 1994). If skin comes in direct contact with such plants, toxic chemical mediums such as histamine and acetylcholine cause dermatitis accompanied by stinging sensation (Cummings and Olsen, 2011; Grauso et al., 2020). Some of them have medicinal value, which can be used for wound drying and healing, soothing cough, and for different medicinal practices (Quan, 1997; Bhusal et al., 2022). Nettle stems are typically four prismatic with opposing small thorned leaves that are elliptical or broadly oval shaped with serrated leaf edges (Cummings and Olsen, 2011). Most nettles are found at an altitude of roughly 1,000 m along highway sides. It grows in marshes, thickets, gravel slopes, gullies, woodlands, and shaded parts of the mountains (Preston et al., 2002).

*Urtica dioica* L. is a perennial herb that belongs to genus *Urtica* also known as large nettle or stinging nettle (He et al., 2012; He, 2012a; He, 2012b). It contains lignified rhizomes and is dioecious and sparsely homozygous, which can reproduce sexually by seeds and asexually by rhizomes. Their flowering period is from July to August, and harvesting period is from August to September (Liu and Sun, 2005; Srutek and Teckelmann, 1998). The species is widely distributed in temperate and tropical regions of the world and is found in Asia, Europe, Africa, and America (He, B. 2012).It grows mostly in shady and humid areas at altitudes of 3,300–3,900 m (Jan et al., 2017; Grauso et al., 2020; Srutek and Teckelmann, 1998; Liu and Sun, 2005; Maričić et al., 2021). Stems and leaves of *U. dioica* are nutrient rich containing large amount of protein and a variety of essential amino acids, which make it as a useful choice for food and herbal treatments (Singh and Sharma, 2011). Furthermore, it has a variety of active ingredients, i.e., *U. dioica* lectins, polysaccharide, and flavonoids, which can produce antioxidant and anticancer effects at certain concentrations by promoting apoptosis of cancer cells and enhancing expression of pro-apoptotic proteins to exert anti-tumor effects (Wang and Pantopoulos, 2011; Abdeltawab et al., 2012; Said et al., 2015). For almost a century, they have been considered as food materials or a part of them having the ability to prevent and treat diseases (Pant, 2019). They are currently being used with remarkable success in various areas such as makeup (Das and Petruzzello, 2015), medicine (Said et al., 2015), healthcare, plant pesticides (Sapkota and Shrestha, 2018), and immune stimulants (Di Virgilio et al., 2015; Grauso et al., 2020; Maričić et al., 2021). It has been reported that the appropriate inclusion of *U. dioica* in animal diet can alleviate gastrointestinal motility disorders in ruminants and improve their immunity, which ultimately improves feed conversion and economic efficiency. At present, scientific research and commercial value of *U. dioica* has once again attracted the attention of European and American countries, Germany, Austria, Finland, the United Kingdom, Lithuania, and other countries for its usage as a whole in animal feed and extraction of its medicinal active ingredients (Li and Yang, 2009; Xie et al., 2008). Some domestic research institutes have also conducted some research on *U. dioica* (Dong and Liu, 2008; Liu et al., 2006; Tian et al., 2006; Ji et al., 2007; Liu et al., 2007; Guo et al., 2005; Dong and Liu, 2009), but it is still in the initial stage. In this review, the current research progress of *U. dioica* at home and abroad is reviewed in order to provide reference for future scientific research and further exploitation of *U. dioica* in China.

## 2 Status of germplasm resources of nettle and its distribution

The classification scheme for *Urtica* is similar to how plants are often categorized in classical taxonomy, which is mostly based on physical traits paired with the environment in which they develop. However, due to the complexity and diversity of *U. dioica* species and relative similarity and confusion in leaf morphologies, it is challenging to express the evolutionary aspects of their qualities using standard classification. Errors may happen if one or more morphological markers are overly relied upon for classification. The classification of urticaria can be carried out by use of ITS sequence analysis (Hsiao et al., 1994; Alvarez and Wendel, 2003). Combining two taxonomic methods for comparative classification can improve the classification of *Urtica* species and deepen our knowledge of their phylogenetic relationships.

### 2.1 Status of germplasm resources of genus *Urtica*

**Figure 1:**
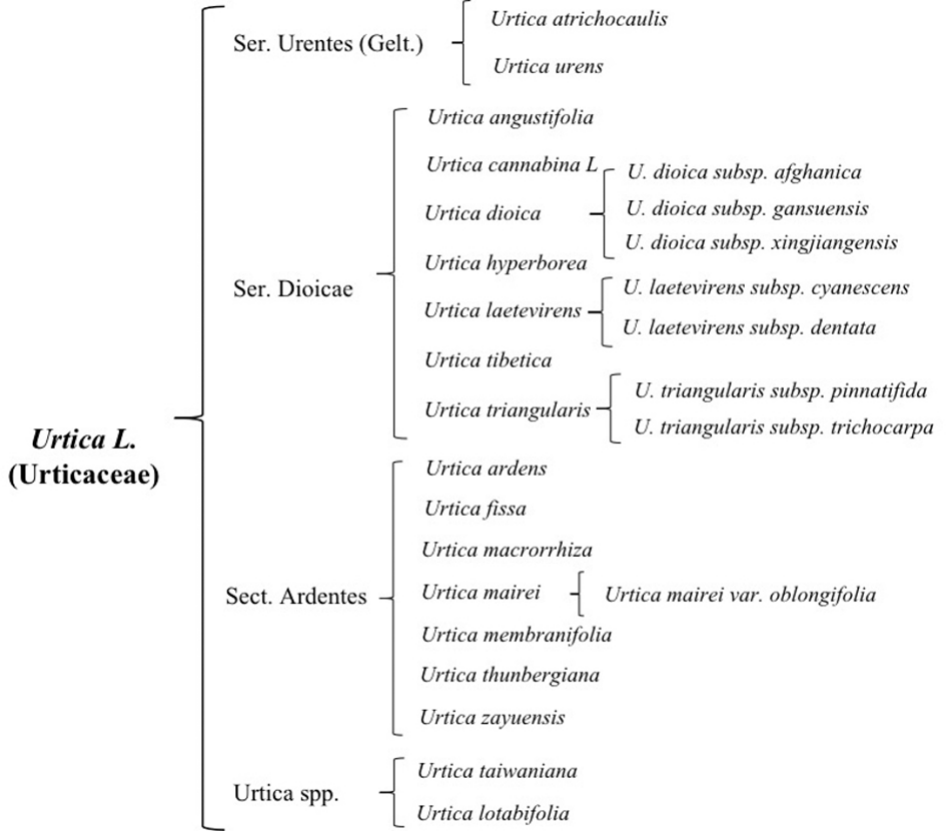
Status of germplasm resources of nettle.

There are 47 genera and approximately 1,300 species of Urticaceae, which are mainly distributed in tropical and temperate regions, almost 35 of which are mainly scattered in temperate and subtropical zone of Northern Hemisphere preferring high temperatures and rainfall (Chen et al., 2003; Su et al., 2018). It is widely distributed in China, Korea, Japan, Mongolia, Russia, and other countries. There are currently 23 species of nettle plants including 16 species, 6 subspecies, and 1 variant, while China has two-thirds of all known species. Approximately 13 species and 3 subspecies of this plant possess best medicinal value and have the ability to dry and heal wound, stimulate blood flow, and relieve pain (Su et al., 2018). Due to its historical usage as a medicinal plant and animal feed, its cultivation has become a general trend (Di Virgilio et al., 2015; Di et al., 2015). On the basis of morphological characteristics, cultivation features, and its germplasm resources, common nettle can be divided into four groups (**Figure 1**); its distribution and growth environment are shown in **Table 1**.

**Table 1:** Distribution and growth environment of nettle.

	Name	Distribution Area	Phenology	Growing Environment
Ser. Urentes(Gelt) C. T. Chen	*Urtica atrichocaulis*	Southwest Region	May–July, July–September	By the roadside at the foot of a mountain, in a valley or by a ditch
	*Urtica urens*	Europe and North Africa	May–July, August–September	By the roadside of the forest edge or next to the residence
Ser. Dioicae C. T. Chen	*Urtica angustifolia*	Northeast, Inner Mongolia, Hebei	June–August, August–September	Mountainous river valley streamside or tableland wet place
	*Urtica cannabina L*	Northwest, Northeast, North China, Sichuan and Inner Mongolia	July–August, August–October	Hilly grasslands or slopes, on the slopes of sand dunes, river floodplains, river valleys, beside streams, etc.
	*Urtica dioica*	Qinghai, Inner Mongolia, Tibet, Xinjiang, Yunnan, etc.	June–July, August–October	Hillside Meadow
	*Urtica hyperborea*	Xinjiang, Tibet, Sichuan and Gansu	June–July, August–September	Alpine gravel land, rock crevices or hillside meadows
	*Urtica laetevirens*	Northwest, Northeast, North China and Southwest China	June–August, August–September	Shady and wet places in the valley under the forest on the hillside
	*Urtica tibetica*	Tibet and Qinghai	June–July, August–October	Hillside Meadow
	*Urtica triangularis*	Qinghai, Sichuan, and Yunnan	June–August, August–October	Wet places in valleys or semi-shady mountain slopes by the side of bushes, houses, etc.
Ser. Ardentes C. J. Chen	*Urtica ardens*	Tibet, China, Yunnan, China, Nepal, and India	July–August, October–November	Underwood
	*Urtica fissa*	Anhui, Zhejiang, Fujian, Guangxi, Hunan, Hubei, Henan, Shaanxi, Gansu, Sichuan, Guizhou, and Yunnan	August–October, September–November	Semi-shady places on hillsides, roadsides or next to houses
	*Urtica macrorrhiza*	Yunnan, Tibet, and Sichuan	July–August, September–October	scrub
	*Urtica mairei*	Tibet, Yunnan, Sichuan, India, Bhutan, and Myanmar	July–August, September–October	Damp place under the forest
	*Urtica membranifolia*	Tibet	May–June, July–August	Underwood
	*Urtica thunbergiana*	Taiwan, Hunan, Hubei, Hebei, China	July–September, August–October	High mountain or low mountain shade and wet place
	*Urtica zayuensis*	Tibet, China, and India	June–July, July–September	Under evergreen broad-leaved forest in gully and valley grasses or grassland on hillside farmland
Urtica spp.	*Urtica lotabifolia*	Shaanxi, Gansu, Sichuan, Guizhou and Zhejiang	August–October, September–November	Mountain slopes, roadsides, or semi-shaded residential areas
	*Urtica taiwaniana*	Taiwan, China		Hillside

#### 2.1.1 Ser. Urentes(Gelt) C. T. Chen

Ser. Urentes includes *Urtica atrichocaulis* and *Urtica urens*, which are mainly distributed in western China (Kregie et al., 2018). Members of this group are mostly quadrangular in shape with spiny hairs and sparse fine hairs or branched pubescence. There are four stipules per node, which are distinctively spaced and contains chromosomes 2n=48 (22, 24, 52). Flowers are monoecious, spikelike, or paniculate. Flowering time is from May to July, while harvesting is done from July to September. Compared to *U. urens*, *U. atrichocaulis* has taller and slender stems having lignified rhizomes (Wang et al., 2012). It grows between 300 and 2,600 m by the roadside of foothills, valleys, or ditches. However, *U. urensis* is an annual herb that grows at a height of 2,800–2,900 m along roadsides or next to the residence (Urtica dioica; Urtica urens (nettle). Monograph, 2007).

#### 2.1.2 Ser. Dioicae C. T. Chen

Ser. Dioicae includes *Urtica angustifolia*, *Urtica cannabina* L, *Urtica dioica*, *Urtica hyperborea*, *Urtica laetevirens*, *Urtica tibetica*, *Urtica triangularis*. Subspecies of *U. dioica* include *U. dioica* subsp. *afghanica*, *U. dioica* subsp. *gansuensis*, and *U. dioica* subsp. *xingjiangensis*. Subspecies of *Urtica laetevirens* Maxim include *U. laetevirens* subsp. *cyanescens* and *U. laetevirens* subsp. *dentata*, while subspecies of *U. triangularis* include *U. triangularis* subsp. *pinnatifida* and *U. triangularis* subsp. *trichocarpa*. 

This group is mostly perennial herbs with lignified rhizomes. Their stems are upright or creeping with exception of *U. hyperborean* whose nettle stems are longer and has sparsely stinging hairs and sparse fine hairs and are typically unbranched or less branched. Flowers are androgynous, more spike-like in shape and a few short to clustered. Some male flowers are in the upper part of the inflorescence, while female ones are in the lower part. *Urtica angustifolia* and *U. dioica* are dioecious, whose flowering period is from June to August and harvesting period from August to October. They grow in mountain valleys and stream sides or shady places on mountain slopes. This group is mainly distributed in northern and western parts of China.

#### 2.1.3 Ser. Ardentes C. J. Chen

Sect. Ardentes includes *Urtica ardens*, *Urtica fissa*, *Urtica macrorrhiza*, *Urtica mairei*, *Urtica membranifolia*, *Urtica thunbergiana*, and *Urtica zayuensis*. Among them, *U. zayuensis* has a variety, which is known as *Urtica mairei* var. *oblongifolia*.

This group mainly consists of perennial herbaceous plants with quadrangular and long rhizomes. It has a fine covering of rough and spiny hairs, which is unbranched or less branched. They are either monoecious or heteroecious that are irregularly mixed only in inflorescences where male and female inflorescences meet or dioecious. Most flowering happens from July to August, while harvesting is from September to November. Suitable growing environment is mostly in thickets, mountain slopes, forest understory, and other shaded and wet places. It is mainly distributed in the western and coastal areas of China.

Among them, *U. thunbergiana* is similar to *U. zayuensis*. However, upon closer inspection, leaves of *U. thunbergiana* are membranous ovate, triangular ovate, or narrowly ovate, axially more pubescent with serrate leaf margins, densely hirsute stems, and subspike-like inflorescences.

#### 2.1.4 *Urtica* spp.

This is an undetermined group of *Urtica* genus, which is mainly distributed in Coastal China and Taiwan area and includes *Urtica lotabifolia* and *Urtica taiwaniana*. They mostly are perennial herbaceous plants (2n=52) having a prism-shaped stem whose height is between 40 and 100 cm. Plants in this group are monoecious and few dioecious as well. Stipules are two per node having connate with panicles. Growing environment is mostly hillside, roadside, or semi-shade wet place of residential areas.

### 2.2 Phylogenetic analysis of chloroplasts of nettles

In most eukaryotes, sequence polymorphism of ITS fragments is very extensive. Therefore, two closely related species usually exhibit notable changes in ITS sequence, indicating recent evolutionary traits. Due to abundant information on loci and variation in loci in the ITS region, it has been confirmed that ITS is an important molecular marker for the study of system and evolution of many angiosperms such as systematic classification of bamboo species by ITS sequence analysis and identification of various Chinese medicinal plants (Xu et al., 2010). ITS is frequently utilized in the study of plant phylogeny due to its accurate reflection towards affinities across genera, families, and species (Thompson et al., 1997; Liu and Zhang, 2012; Alvarez and Wendel, 2003; Li et al., 2016).

**Figure 2:**
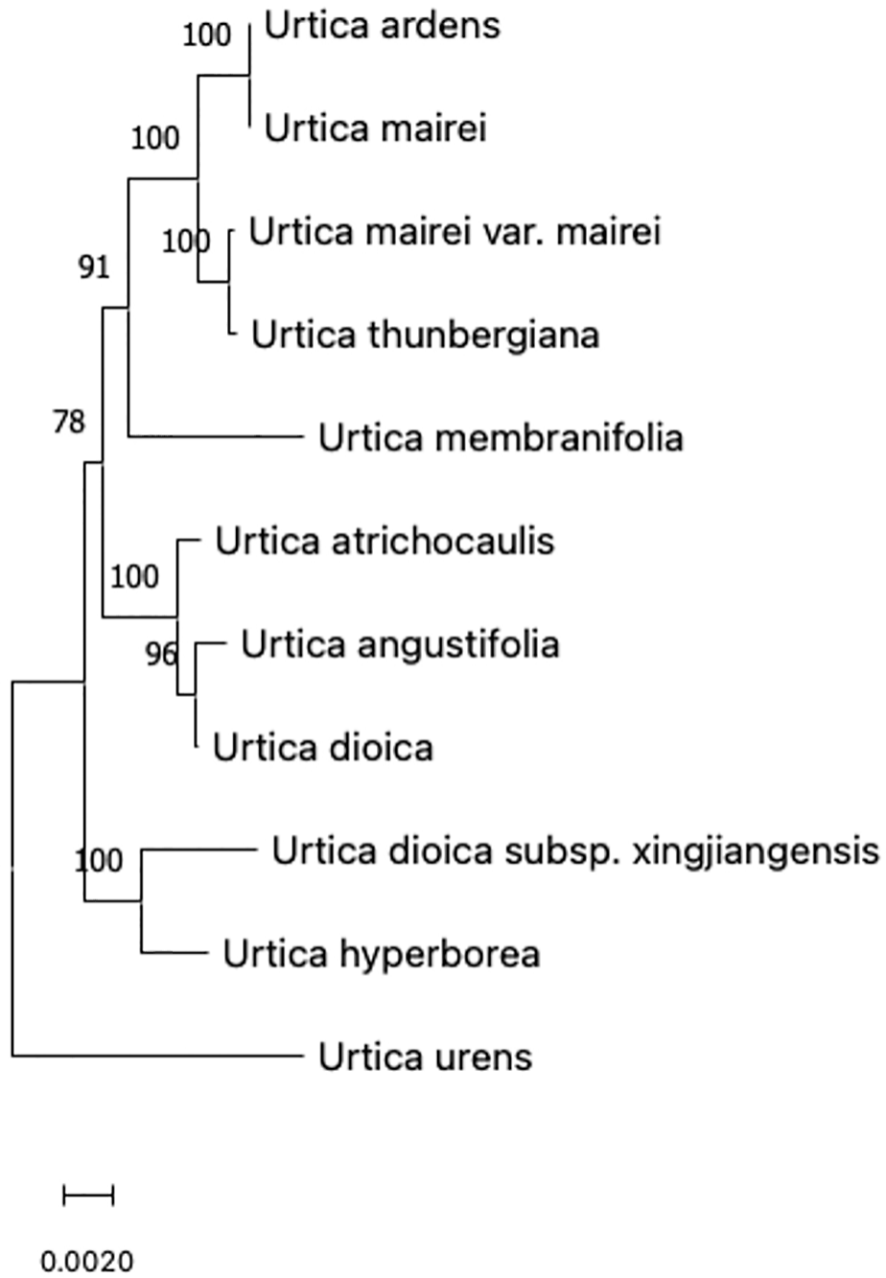
Phylogenetic tree of 11 species of *Urtica* spp. constructed based on NJ method.

A comparison of ITS sequences of GenBank was done. GenBank sequence registration numbers of CP-chloroplast genome, ITS (ITS1-5.8S-ITS2), and nrDNA-nuclear ribosomal DNA (18S-ITS1-5.8S-ITS2-26S) are shown in **Table 2**. ITS sequences were completely matched with MEGA, and genetic distances were calculated. Then, a phylogenetic tree was constructed using the neighbor-joining matrix method, and morphological markers were clustered as shown in **Figure 2**.

**Table 2:** Gen Bank sequence registration numbers of *Urtica* spp.

	Species name	Each DNA region GenBank registration number	
		CP	ITS/nrDNA
1	*Urtica angustifolia Fisch*	OM761939	OM892788
2	*Urtica ardens*	OM761941	OM892790
3	*Urtica atrichocaulis*	OM761942	OM892791
4	*Urtica dioica L.*	OM761947	OM892796
5	*Urtica dioica* Linn. subsp. *xingjiangensis* C.J.Chen	MT465760	OM892795
6	*Urtica hyperborean* Jacq	OM761949	OM892798
7	*Urtica mairei* Levl	OM761952	OM892802
8	*Urtica mairei* Levl. var. *oblongifolia* C.J. Chen	OM877284	OM892803
9	*Urtica membranifolia*	OM761953	OM892804
10	*Urtica thunbergiana* Hand.-Mazz	OM761957	OM892809
11	*Urtica urens* L.	OM761959	OM892810

The evolutionary history was inferred using the neighbor-joining method to form an optimal tree (Saitou and Nei, 1987). The percentages of replicate trees in which associated taxa clustered together in the bootstrap test (500 replicates) are shown above the branches (Felsenstein, 1985). The tree is drawn to scale with branch lengths in the same units as those of the evolutionary distances used to infer phylogenetic tree. The evolutionary distances were computed using p-distance method and are in units of number of base differences per site (Nei and Kumar, 2000). This analysis involved 11 nucleotide sequences. Codon positions included were 1st+2nd+3rd+non-coding. All positions containing gaps and missing data were eliminated (complete deletion option). There was a total of 5,764 positions in the final dataset. Evolutionary analyses were conducted in MEGA11 (Stecher et al., 2020; Tamura et al., 2021).

According to the clustering of chloroplast gene polymorphism, results were basically consistent with the traditional classification but not completely consistent with 11 species of Urticaria that were divided into two branches with 100% support. Because *U. urens* L has special characteristics in the length of its growth cycle, it is an annual herb, while other species are perennial herbs. It may be due to this difference in the growth cycle that *U. urens L.* is separated first in the first branch and divided into a large branch by other species, indicating that this kind of urticaria is far related to other species. However, it can be seen from the figure that *Urtica dioica L.* and *U. angustifolia* did not gather on the same branch with other kinds of urticaria in the traditional classification. The phylogenetic tree showed that *Urtica dioica L.* and *U. angustifolia* were far away from *U. hyperborea* and *U. dioica* subsp. *xingjiangensis. Sect. Ardentes* under urticaria clustered in one branch, which was consistent with the traditional classification results.

The genetic distance of 11 species of urticaria ranged from 0.008 to 0.023, while that of most species was <0.02, indicating that 11 species of urticaria were closely related. Among them, genetic distance between *U. dioica L.*, *U. angustifolia*, and *U. atrichocaulis* was 0.0014 and 0.0017, which shows that the relationship between different strains of nettles and two species of nettles is very close.

## 3 Characteristics of *Urtica dioica*

Common field weed *U. dioica L.* possesses large population density and remarkable potential for reproduction and regeneration. Its seeds, stems, leaves, and roots have a tremendous developmental and usage potential. Planting *U. dioica* L. can enhance the ecological environment in addition to providing good economic benefits (Di et al., 2014). The key characteristic of the germplasm of *Urtica dioica* L. that stands out most is its tolerance to shade, drought, and barrenness. Its ability to adapt shady areas and drought conditions during cultivation makes it even more valuable. This ability is attributed to an increase in water distribution in the roots and a decrease in water distribution in stems and leaves (Liu et al., 2007). Moreover, during drought conditions, *U. dioica* reduces light uptake that ensures carbon assimilation through light energy conversion efficiency.

At present, it seems that *U. dioica* can be grown in an environment having moisture content of 60%–100% (particularly 85%). During drought conditions, it increases its resilience to water stress by altering the concentration of chemicals involved in metabolism and synthesis (Liu, 2007). The ratio of above-ground water content to total water dramatically drops as the severity of drought stress rises (soil water content decreases), whereas the ratio of root water content to total water greatly increases, which increases specific gravity of root and root to shoot ratio.

### 3.1 Drought resistance

Drought resistance is a characteristic possessed by *U. dioica* L. due to following reasons: thin leaves and leaf cuticle, low percentage of epidermal cells, only one layer of cells in fenestrated tissue, loose cell arrangement, less thickness, and upward arching of stomatal guard cells (Liu, 2007).

In addition to the built-in property of drought resistance in plants, environmental factors can also significantly influence drought resistance. The most significant environmental factor influencing crop growth, development, and water usage efficiency is the level of soil moisture. Likewise, survival, plant height, leaf area, and internal water distribution of *U. dioica* L. are generally affected by soil moisture content (Vogl and Hartl, 2003). Moisture stress is also closely related to reactive oxygen species accumulation. Different oxidative enzymes (SOD, CAT, and POD), osmoregulatory substances (Pro, soluble proteins, and sugars), permeability, and peroxides (MDA) can be significantly affected by soil moisture content in *U. dioica* L. (Wang and Li, 2001). It was reported that the plants developed under normal water supply (60%) compared to plants during drought stress have more water content in leaves, whereas water content of plants in drought conditions increased in roots compared to stem and leaves. Thus, *U. dioica* L. fights drought damage by allocating more water to roots and less to stem and leaves. Due to the resistance of *U. dioica* L. to drought, appropriate irrigation and watering are sufficient to meet its water needs during growth (Vogl and Hartl, 2003; Akgil, 2013; Bisht et al., 2012). Moreover, under drought conditions, *U. dioica* L. mainly allows roots to preferentially absorb water and mineral elements, forming a root redundancy phenomenon as a response to water stress. Similarly, the growth of *U. dioica L.* was not even slowed down by moderate spring environment of adequate precipitation and by the summer’s plentiful precipitation, which produced 56 mm of rainfall (Bacci et al., 2009). Moisture content also affects photosynthesis, transpiration rate, and stomatal conductance of plants. It has been observed that range of light intensity usage was widest and adaptation to light environment was strongest when soil moisture content was 85%. Moreover, net photosynthetic rate was at its peak, and it lowers down when temperature change occurred in either direction.

### 3.2 Shade tolerance

Under shade conditions, maintaining a high light energy use efficiency is essential for plant growth and many related biochemical, physiological, and morphological processes. Therefore, changes in plant photosynthesis curves under shade resulting in light deficiency are an important response to plant light energy use efficiency. Net photosynthetic rates were observed to vary in different experiments as *Urtica dioica L.* leaves display a bimodal daily fluctuation in summer while a unimodal daily variation in autumn (He et al., 2010; He and Liu, 2012a; 2012b). Under shading conditions, light compensation point, light saturation point, net photosynthetic rate, dark respiration rate, and chlorophyll a/b decreased, while chlorophyll content, light energy utilization, PSII primary light energy conversion efficiency, and potential activity increased, thus improving the growth and development of *U. dioica* L. under low light conditions (Liu et al., 2007). Additionally, ratios of total chlorophyll, chlorophyll a, and chlorophyll b concentrations in its leaves were observed to be on a significantly higher side in a shady environment. Thus, it can be stated that *U. dioica* L. has a high adaptability and can self-regulate with the external environment; thus, *U. dioica* L. has a strong shade tolerance.

Under moderate water stress, photosynthetic capacity of *U. dioica* enhances a significant effect on chloroplast pigment content due to increase in range of light intensity utilization and adaptation to a light environment (He et al., 2012). The ratio of chlorophyll a to b reveals how sensitive the plant is to photochemical reactions, with chlorophyll a primarily carrying out photochemical processes and chlorophyll b primarily absorbing light energy. Concentrations of *U. dioica*’s chlorophyll a, chlorophyll b, total chlorophyll, and carotenoid compounds were highest under 85% soil moisture content, while the ratio of chlorophyll a/b changed with changing soil moisture content and decreased with increasing shade, suggesting that *U. dioica* has sensitivity towards water stress. When soil water deficit inhibits plant growth, the conversion rate from chlorophyll b to chlorophyll a is significantly accelerated and the ratio of chlorophyll a/b increases with decreasing water content, which indicates that chlorophyll b is more fragile and can only be reduced to increase chlorophyll a. Thus, it can be stated that *U. dioica* has a good drought response mechanism (Quan, 1997; Liu and Sun, 2005).

### 3.3 Barren tolerance

*Urtica dioica* L. has good ecological adaptability mainly due to its developed underground rhizomes, which can easily survive at low temperatures (Akgil, 2013). It grows rapidly in areas with loose soil texture and rich organic matter such as nitrogen and phosphorus, while it has poor waterlogging resistance and mostly grows in sandy loam.

*Urtica dioica* L. is a nitrophilic plant that likes to grow in the soil having pH 5.6–7.6 (Di et al., 2014). It is a suitable choice for phytoremediation due to its ability of growing normally in overfertilized or nitrogen-rich soils. However, the effect of excess nitrogen on its fiber content needs to be further explored (Adler et al., 2008). By incorporating rich mineral materials into the soil during the apoptosis phase, the planting of *U. dioica* L. can successfully prevent soil degradation processes including soil erosion and thinning of soil layer (Di et al., 2014). Moreover, *U. dioica* L. has a large advantage over other weeds when fighting for water and nutrients in the soil during planting, so it can reduce usage of chemical herbicides (Bacci et al., 2009).

In early stage of planting *U. dioica* L., field management should be strengthened. In the process of agricultural production, cultivation row spacing can be controlled at 100–150cm, planting rows can be controlled between each other, which is conducive to mechanized weeding (Di et al., 2014). Stems and leaves of wild *U. dioica* L. can easily be damaged by fungi, caterpillars, aphids, and starscream, which can be effectively controlled by diphenyltriazolol, methyl trobuzine, and organic sulfur pesticides (Shattock, 2005).

The germplasm specificity of *U. dioica* mainly includes shade tolerance, drought tolerance, and barrenness. It is mainly affected by the dominant ecological factors, such as light, moisture, and soil. The analysis and determination of these dominant ecological factors often play a key role in the success of seed introduction.

## 4 Nutritional and active ingredients

### 4.1 Nutritional profile

Upon feeding at proper inclusion rate, *U. dioica* L. can positively affect the growth and development of livestock due to its nutrient-rich profile. It contains calcium, magnesium, potassium, phosphorus, iron, and several other minerals (Said et al., 2015). Likewise, it has low sodium content, which is advantageous for growth and development of an individual. Protein and essential amino acids are present in abundant quantity in roots, stems, leaves, fruits, and chelonian, out of which 70% proteins are digestible (Zhang and Zhao, 2008). It was observed that the protein content of leaves before flowering was higher compared with after flowering, which was 27.4% and 20.7%, respectively. Moreover, it also contains almost 18 different amino acids, including 16 in free form (Han et al., 2001).

*Urtica dioica* L. is a nitrophilic plant that grows rapidly in loose soils rich in organic matter such as nitrogen and phosphorus and therefore has the highest nitrogen content of 587 mg/ml in its sap. Under shade, it was found that the concentration of total chlorophyll, chlorophyll a, and chlorophyll b in *U. dioica* L. leaves grew dramatically, which results in high vitamin C, folic acid, vitamins A and E content, and several other nutrients. Its leaves contain 140 and 300 mg of carotenoids and 1,000–2,000 mg of vitamin C on dry matter basis (Zhang and Zhao, 2008). It contains rich quantity of minerals, which helps in balancing Cu/Zn ratio in the human body that can prevent cardiovascular and other disorders (Han et al., 2001).

### 4.2 Active ingredients

Active ingredients in plants are chemical components that can promote or inhibit growth and development of living cells. Many active ingredients found in different parts of *U. dioica* include flavonoids, organic acids, phenols, phenylpropanoids, polysaccharides, and many other compounds (Bhuwan et al., 2014).

*Urtica dioica* agglutinin (UDA) is a protein having red blood cell adhesion capabilities isolated from *Urtica*’s roots (Hu, 2006). It is a single-chain polypeptide composed of 11 exogenous lectins and 80–90 amino acids that has anti-inflammatory and antiviral properties (Van et al., 1988). This protein has the ability to prevent arachidonic acid metabolism in viruses and cause T-lymphocytes to create cells in a particular manner to enhance immunity.

One of *U. dioica’*s key active ingredients is crude polysaccharide, which can inhibit adjuvant arthritis in rats by affecting various stages of the disease. This helps in minimizing its toxic effects on the body (Zhang, 2006). Currently, several flavonoids including flavonols and glycosides have been extracted from whole grass and flowers of *U. dioica* L. (Liu, 2007). Several other compounds like isorhamnetin, quercetin, and naphthol have significant physiological and biochemical effects like lowering blood pressure, lowering blood lipid levels, anti-thrombus, and cardiovascular protection, and antiviral, antioxidant, anti-tumor, anti-inflammatory, and antibacterial properties (Zhang et al., 2009).

The components and effects of *U. dioica* are shown in **Table 3**.

**Table 3:** Components and effects of *Urtica dioica*.

Plant Parts	Active Ingredients/Nutrients	Physiological Effects	Treatment of Diseases
Dioecious nettle whole herb and flowers	Flavonoids	Medicinal anti-inflammatory activity, biological activity, and pharmacological action	Treatment of arthritis and rheumatism
*Urtica dioica* root extract	Phenols	Release nitric oxide to relax blood vessels, open potassium channels, and weaken vasoconstriction	Treatment of hypertension
	Organic acids	Release nitric oxide to relax blood vessels, open potassium channels, and weaken vasoconstriction	
	Proteins and polysaccharides	Inhibits cell proliferation and epithelial cell growth factor binding to tumor cell receptors	Inhibits arthritis, prostate, skin diseases, etc.
Aqueous extract of *Urtica dioica*	Organic acids	Inhibition of binding of sex hormone-binding globulin to human prostate cell membranes	Treatment of heart disease
Stinging hairs on the surface of plant leaves and stems and leaves	Other Ingredients	Inhibition of excessive cytokine growth	Treatment of rheumatoid arthritis, allergic rhinitis

## 5 Physiological effects of active substances on animals

Flavonoids present in *U. dioica* L. are derivatives of chromophen or chromophen, such as quercetin, hypericin, and kauniol, which have a basic skeleton C6–C3–C6 consisting of two aromatic rings connected by a central three-carbon chain (Liu and Sun, 2005). Flavonoids, steroids, coumarins, lignans, and other active substances in different parts of *Urtica* have the ability to inhibit benign prostatic hyperplasia and cancer cells division. They also exhibit anti-rheumatic, hypoglycemic, analgesic, anti-inflammatory, and antioxidant properties (Quan, 1997; Srutek et al., 1998; Jakubczyk et al., 2015). These compounds have significant therapeutic effect on benign prostatic hyperplasia, rheumatism, arthritis, and other diseases (Di et al., 2015; Zhang et al., 2009).

Flavonoids are polyphenolic metabolites with a remarkable antioxidant activity. Excess free radicals in the human body can cause cellular damage, leading to aging, tumors, inflammation, diabetes, atherosclerosis, etc. (Singh et al., 2015). It has been observed that *U. dioica* has shown significant therapeutic effect on allergies, kidney stones, burns, anemia, rashes, bleeding, and diabetes (Singh et al., 2012). Quercetin present in *Urtica dioica* L. has anticancer effects and can form resonance stabilized phenoxy radicals, which can directly hunt peroxyl radicals and inhibit cell damage caused by reactive oxygen species at certain concentrations (Procházková et al., 2011; Gabriele, 2015).

Although OH^−^ is a very active free radical in the body, it is also quite damaging to the body. At present, it has been found that a variety of antioxidants have significant effects on the removal of superoxide anions; however, OH^−^ cannot be removed. Studies have shown that flavonoids have significant effects on OH^−^. Furthermore, its antioxidant ability against oxide and hydroxyl free redicals is stronger than that of standard antioxidant, i.e., vitamin E (Mohammad et al., 2017).

**Figure 3:**
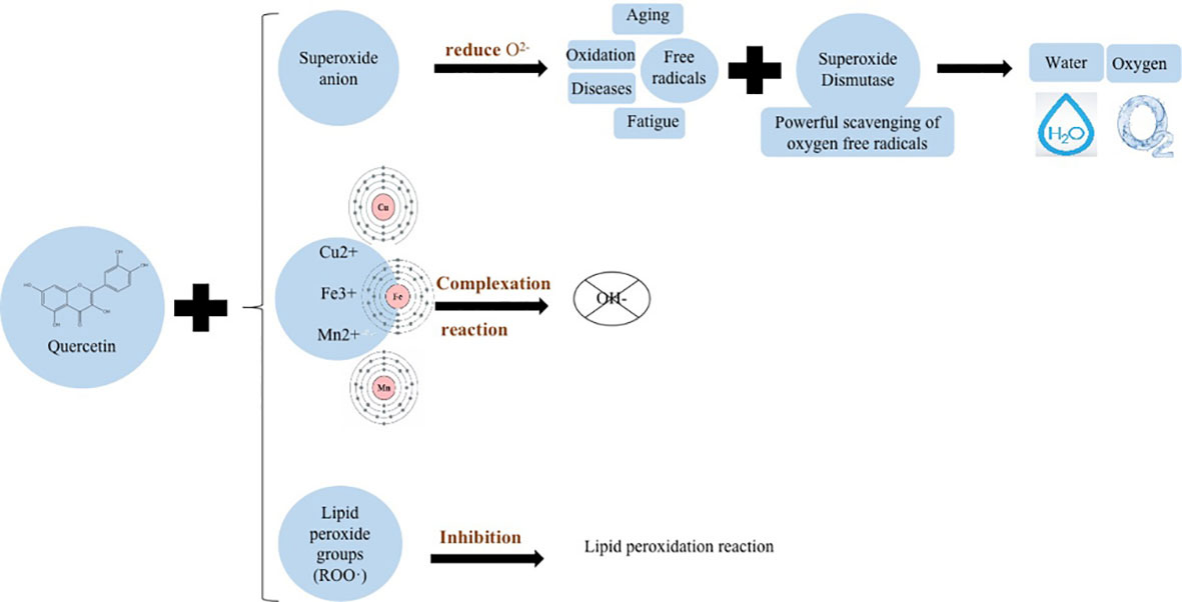
Anti-free radical effect of quercetin.

Flavonoid compounds like quercetin primarily inhibit formation of free radicals in three ways: (1) by directly binding to superoxide anions to reduce oxygen radical production, (2) by binding to Cu^2+^, Fe^3+^, and Mn^2+^ to inhibit hydroxyl radical OH^−^ formation, and (3) by reacting with lipid peroxyl radicals to inhibit lipid peroxidation as shown in **Figure 3** (Du et al., 2018). Nettle treatment with carbon tetrachloride (CCl_4_) decreases lipid peroxidation and increases the activity of antioxidant defense system in rats (**Figure 1**), which ultimately prevents liver damage. Thus, quercetin has antioxidant and anti-free radical properties that enable it to combat cancer by inhibiting cell death caused by lipid peroxidation.

Oxidative stress causes inhibition of osteoblasts differentiation, which might result in osteoporosis. Quercetin pairs are able to upregulate the expression of antioxidant response genes and proteins such as NF-E2-related factor 2 (Nrf2), ERK1/2, and NF-κB. It can be suggested that antioxidant response of osteoblasts can be used to prevent osteoporosis (Messer et al., 2015). Numerous oxidative disorders are caused by reactive oxygen and reactive nitrogen species that are created by the oxidation of activated neutrophils and macrophages. Kim et al. (2013) stimulated mouse macrophages RAW264.7 by using yeast polysaccharide and adding a certain concentration of quercetin to cells. Results had shown that quercetin was able to inhibit the yeast-stimulated RAW264.7 cells from the inhibitor protein α of nuclear factor κB. Moreover, quercetin can also inhibit phosphorylation and degradation of NF-κBα (Iκbα) inhibitor in yeast-stimulated RAW264.7 cells, which leads to inhibit expression of i-NOS to produce antioxidant effects.

**Figure 4:**
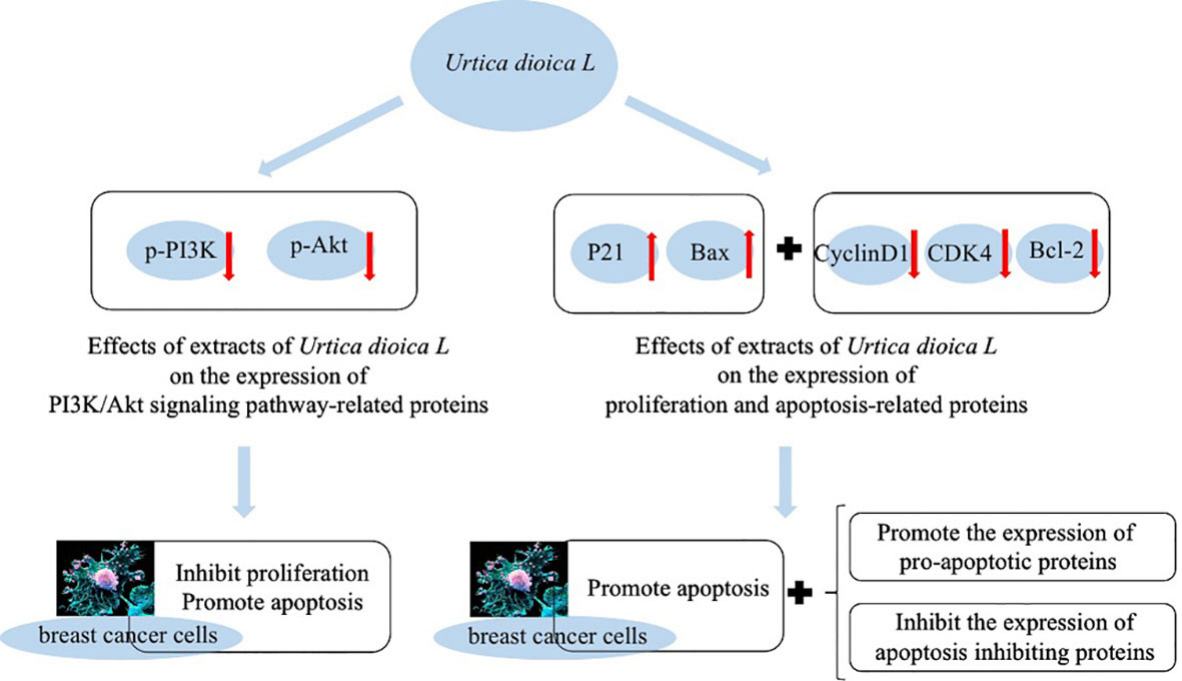
Inhibition of cancer cell proliferation by *Urtica dioica* L.

Besides having antioxidant capacity, flavonoid compounds such as quercetin can also affect signal transduction in tumor cells. They have low toxicity to normal cells and can target multiple signals, thus effectively killing tumor cells and can effectively inhibit the growth of cancer cells and induce apoptosis (Liu and Sun, 2005). It has also been found that *U. dioica* L. extract exerts some inhibitory effects on proliferation of breast cancer cells by blocking them in G0/G1 phase, which may be related to the inhibition of PI3K/AKT signaling pathway. Expressions of p-PI3K and p-AKT were significantly decreased in the cells of *U. dioica* L. group, while BAX protein expression was significantly increased, which indicates pro-apoptotic effect of *U. dioica* L. on breast tumor cells (**Figure 4**) (Ji et al., 2021).

Meanwhile, Ji et al. (2021) also treated breast cancer cells MCF-7 and MDA-MB-231 with drug and without drug to observe the expression of proteins related to cell proliferation and apoptosis. They reported that the expression of P21 and Bax proteins was significantly increased, while the expression of CyclinD1, CDK4, and Bcl-2 proteins was significantly decreased in the *U. dioica* L. group compared with the control group. It also indicates that *U. dioica* L. extract has anti-tumor effects that can promote apoptosis and expression of pro-apoptotic proteins in breast cancer cells.

It has also been found that adding 5 and 10 mg/ml extract of *U. dioica* L. to breast cancer cells causes the suppression of cell proliferation by MCF-7 and MDA-MB-231 and G0/G1 phases of cell cycle. It increases apoptosis rate dramatically and decreases number of cell clones. We can say that the extract of *U. dioica* L. can successfully induce breast cancer cells’ apoptosis, inhibit tumor cells in G0/G1 phase, and reduce growth of cancer cells (Ji et al., 2021).

## 6 Status of usage as fodder

### 6.1 Health performance

Nettle has been extensively included in livestock production due to its abundant nature, high nutritional value, and potent pharmacological effects. *Urtica dioica* L. can be fed directly or in dried form such as hay, grass meal, or silage as high-quality animal forage. Bisht et al. (2012) reported that intake of protein and vitamins was found to be significantly increased, and use of green forage was significantly decreased when young stems and leaves of *U. dioica* L. were used as forage in animal diet. Immunity in pigs was shown to be greatly increased when nettle was added to their meal, and feed conversion rate was also found to be significantly improved.

Similarly, the sap of *U. dioica* had a promoting effect on isolated small intestinal motility of mice and gastrointestinal absorption in sheep and mice. It also has some mitigating effects on digestive tract disorders in ruminants. Humphries and Reynolds (2014) used nettle instead of ryegrass silage in milk rations to stabilize rumen pH in cows and found it beneficial to rumen health and not affecting milk production.

The entire nettle plant is nutrient rich containing a variety of vitamins, organic acids, tannins, and other compounds. However, its stems and leaves contain some irritant acidic substances such as formic acid (anthranilic acid) and acetic acid. If ruminants consume a lot of young nettle, a lot of acidic substances build up in the rumen resulting in acidosis and decline in feed intake. Thus, it must be used with proper inclusion levels. Nettles are often used as feed for pigs, which are omnivores with a high acid tolerance and are able to convert ingested organic acids into body fat themselves. The main form often offered is through the addition of dried and crushed nettles to the diet. Because of the high protein content of nettles, direct silage can lead to nutrient loss, so it is mostly used as a mixed silage with additives. As nettle stem bark contains some harsh acidic substances such as formic acid, acetic acid, and caseic acid, most of them are used in ruminant feeding after flowering period. If ingested in large quantities during the early period, organic acids in stem bark will accumulate in rumen and lead to acidosis. In ewes, nettle was added as a forage during gestation, which significantly increased milk production after parturition and weight in lambs. Moreover, resistance in both ewes and lambs against diseases was improved. However, acute gastric dilatation occurred because of rough feeding of camels and a large amount of nettles consumed during the budding and re-greening period. Similarly, Zhang and Zhao (2008) found a significant increase in milk production, rapid lamb growth, and a significant decrease in disease rate in pregnant ewes upon feeding nettle as a forage. Nettle was found to be an ideal feed ingredient for raising beef calves and heifers throughout fodder scarcity time period by having high-quality nutritional and flowering stages.

### 6.2 Production performance

*Urtica dioica* L. can reduce feed cost without compromising on animal production performance when it is used as a forage source. Moreover, it should be fed in moderation to avoid acidosis caused by overfeeding of animals. Liu et al. (2010) tested the production performance of laying hens by adding nettle to the diet of 51-week-old Hyland brown laying hens and found that egg production rate was significantly increased with 0.1% inclusion of nettle. Furthermore, specific gravity, yolk color, shell thickness, and Hastelloy units of eggs were significantly higher when 0.15% nettle was added, while cholesterol content in yolk was significantly decreased. It has also been observed that the inclusion of nettle stems and leaves at various dietary levels in broiler diets improved broiler performance with a significant increase in broiler growth rate and body weight (Bekele et al., 2015).

Wang and Jiang (2002) reported that adding nettle paste in layer diet improved not only the hatching rate but also the production performance and egg yolk color. Moreover, it also reduced breeding cost, thus significantly improved the economic efficiency of laying hens (Loetscher et al., 2013). It has also been observed that the addition of nettle in laying feed increased egg production rate and significantly decreased disease rate of young poults. Ma (2014) added 1%, 3%, and 5% nettle to broiler diets for 60 consecutive days and found that survival rate, carcass weight gain, and feed to weight ratio were significantly higher at all inclusion levels compared with conventional feed. Furthermore, the market price of live chickens plus the cost of grain savings was significantly higher in all three experimental groups compared with control group at $5.1, $7.2, and $5.9, respectively. Thus, it can be stated that adding nettle to broiler diets can improve poults immunity, feed conversion rate, and profitability.

### 6.3 Resource development for usage as fodder

According to the Dictionary of Chinese Materia Medica, stinging acid substances are present in nettle, which can lead to ruminal acidosis. In cattle, sheep, camels, and other herbivores, nettles are suitable to be used after the flowering period and in hay form during winter and spring. Thus, if we want to use nettle for treatment of gastrointestinal dysfunctional diseases or as a feed ingredient, then it should be used after flowering to optimize proper inclusion levels, so it can play its role in promoting digestion, absorption, and nutrition. In winter and spring during the calving season, supplementing nettle hay not only solve green forage problem but also improve immunity of livestock, disease prevention, and postpartum recovery of female animals. It can prove to be an ideal animal feed ingredient with high protein, fat, fiber, and fatty acids.

Water content in above-ground parts under conditions of drought stress was found to be significantly decreased as a percentage of the total, while water content of roots increased significantly as a percentage of the total. Additionally, root to crown ratio also significantly increased at the same time according to germplasm specificity of *U. dioica*. Similarly, it was discovered that the leachate content of *Urtica dioica* roots harvested from late autumn to early spring was the highest, and the leachate content of roots harvested in early spring and winter was high, while it is low when harvested in summer and early autumn. Thus, harvesting from late autumn to early spring, i.e., between November of the first year and March of the following year, was considered as the best time to harvest *U. dioica* roots.

Due to germplasm specificity, nutrient content, and active ingredients present in *U. dioica* L., it is gradually being used as a protein-rich ingredient in animal feed. It can effectively promote production performance, health performance, gastrointestinal digestion, and absorption. In the current context of shortage of protein feed resources, it can fulfill this gap. However, its stimulating substances and mechanism of action need to be explored in depth.

In conclusion, nutrient characteristics of various nettles, type and concentration of active substances, and their effects are different. Therefore, it is necessary to select the appropriate type of nettle by keeping in mind specific characteristics and their effects on animal health, proper inclusion levels in animal diet, anti-nutritional factors, and chemical substances present in it. For this reason, in the future, it is necessary to make a deeper exploitation of nettle in order to alleviate the shortage of protein feed resources and to find effective active substances of alternative resistance.

## Author contributions

YZ and XZ conceived the idea of the review and prepared the initial outline and wrote the first draft. MZ, JZ, JW, and XY gathered the literature and contributed in writing the different sections. MZ, MW, and WL provided the technical guidance and editing support. All authors contributed to the article and approved the submitted version.
